# p53 Aggregates Penetrate Cells and Induce the Co-Aggregation of Intracellular p53

**DOI:** 10.1371/journal.pone.0069242

**Published:** 2013-07-03

**Authors:** Karolyn J. Forget, Guillaume Tremblay, Xavier Roucou

**Affiliations:** Département de Biochimie, Faculté de Médecine et des Sciences de la Santé, Université de Sherbrooke, Sherbrooke, Québec, Canada; Ludwig Maximilians University Munich, United States of America

## Abstract

Prion diseases are unique pathologies in which the infectious particles are prions, a protein aggregate. The prion protein has many particular features, such as spontaneous aggregation, conformation transmission to other native PrP proteins and transmission from an individual to another. Protein aggregation is now frequently associated to many human diseases, for example Alzheimer’s disease, Parkinson’s disease or type 2 diabetes. A few proteins associated to these conformational diseases are part of a new category of proteins, called prionoids: proteins that share some, but not all, of the characteristics associated with prions. The p53 protein, a transcription factor that plays a major role in cancer, has recently been suggested to be a possible prionoid. The protein has been shown to accumulate in multiple cancer cell types, and its aggregation has also been reproduced *in vitro* by many independent groups. These observations suggest a role for p53 aggregates in cancer development. This study aims to test the «prion-like» features of p53. Our results show *in vitro* aggregation of the full length and N-terminally truncated protein (p53C), and penetration of these aggregates into cells. According to our findings, the aggregates enter cells using macropinocytosis, a non-specific pathway of entry. Lastly, we also show that once internalized by the cell, p53C aggregates can co-aggregate with endogenous p53 protein. Together, these findings suggest prion-like characteristics for p53 protein, based on the fact that p53 can spontaneously aggregate, these aggregates can penetrate cells and co-aggregate with cellular p53.

## Introduction

Many human neurodegenerative and non-neurodegenerative pathologies are related to protein aggregation: these disorders belong to a large family of protein ‘conformational diseases’, which includes, most notably, Alzheimer’s disease and prion diseases but also Parkinson’s disease, Amyotrophic lateral sclerosis, chronic pancreatitis, and amyloid A amyloidosis. In these diseases, insoluble protein aggregates accumulate inside or outside the cells. The propagation of protein aggregates has been well studied; particularly in prion diseases where it has been shown that, *in vitro* and *in vivo*, exogenous prion protein (PrP) aggregates can induce misfolding of soluble cellular PrP^c^ into aggregates, termed PrP^Sc^ [1–3]. Since then, many groups have shown the same phenomena with Amyloid-β (Aβ) in Alzheimer’s disease, Tau and α-synuclein in Parkinson’s disease, SOD1 and TDP-43 in amyotrophic lateral sclerosis and Huntingtin in Huntington’s disease [1,4]. Oftentimes, the terms prion-like or prionoid are used to describe proteins that show similar characteristics to prions, such as molecular recruitment and misfolding induction, and intercellular transmission.

A fairly recent candidate for prion-like characteristics is p53 protein, a transcription factor whose function is lost in more than 50% of cancers [5,6]. Many reasons led to the consideration of p53 as a potential prion-like protein. First, it is widely known that p53 has limited structural stability, due in part to the unstructured nature of its N-terminal domain [7]. In fact, different groups have shown that the p53 transactivation (amino acids 1-92), central or core (amino acids 93-312) and tetramerization (amino acids 313-393) domains can all misfold and form fibrillar aggregates *in vitro* in mild denaturing conditions [8–10]. Also noteworthy is the low thermodynamic stability of the core domain, a domain targeted by more than 90% of point mutations that inactivate p53 in cancer [11,12]. Secondly, p53 accumulation in the form of aggregates has been observed in the perinuclear region of several tumor cell lines expressing endogenous p53 mutants, including MOG-G-CCM astrocytoma, HT-1376 bladder carcinoma, Detroit 562 pharynx carcinoma and 1301 T-cell leukemia [13]. Some human neuroblastoma tumors overexpressing wild type p53 also display large p53 positive cytoplasmic protein aggregates [14,15]. Recently, studies have confirmed the co-aggregation of wild-type and mutant p53 in breast cancer and colorectal cancer tissues [13,16]. Finally, dominant-negative activity of p53 missense mutants results from mutant-induced co-aggregation of wild-type p53 in cells co-transfected with mutant and wild-type p53 [13].

Together, these observations suggest an implication for misfolded and aggregated p53 in some cancers and support the hypothesis that p53 aggregates could display prion-like activity. Transmission of the misfolded conformation is a critical feature in the propagation of prion-like proteins [3,17]. Yet, whether p53 aggregates can penetrate the cells plasma membrane and interact with intracellular soluble wild-type p53 to propagate their misfolded conformation is unknown.

Here we report that recombinant exogenous wild-type p53 aggregates generated *in vitro* can penetrate cells by macropinocytosis and induce aggregation of endogenous wild-type p53. This result reveals a key feature of p53 aggregates and supports their prion-like character.

## Material and Methods

### Cloning of p53^GFP^, p53 and p53C (core domain, amino acids 93-393)

Human p53 was amplified by PCR using Addgene plasmid 11770 [18] as a template and forward 5′-gggccatatggaggagccgcagtcagatccta-3′ and reverse 5′-gtggatcctcagtctgagtcaggcccttctgtct-3′ primers. p53C was amplified by PCR using forward 5′-gggccatatgtcatcttctgtcccttcccagaaaacc-3′ and reverse 5′-gtggatcctcagtctgagtcaggcccttctgtct-3′ primers The PCR products were introduced in the NdeI and BamHI restriction sites of pet28a. Primers were purchased from IDT. All constructs were sequenced in both orientations.

### Purification, aggregation and labeling of p53 and p53C

The plasmid pet28a containing the cDNA of human N-terminally His-tagged p53 or p53C was transformed into *Escherichia coli* strain BL21 λ DE3. The resulting bacteria were grown at 37 °C to an OD_600_= 0,6 before overnight induction at 22°C with 0,5 mM isopropyl β-D-thiogalactosidase (IPTG). After induction, cells were harvested by centrifugation, incubated 30 min on ice in lysis buffer (50 mM NaH_2_PO_4_, 1 M NaCl, 40 mM imidazole) and 1 mg/mL lysozyme then sonicated 3 times at 30% amplitude for 30 s (Fisher Scientific). The lysate was centrifuged at 10,000 g 30 minutes and supernatant was incubated 1 h at 4 °C with HisPur Ni-NTA resin (Thermo Scientific). Purification was performed using disposable chromatography columns (Bio Rad): the resin was washed four times using lysis buffer containing 100 mM imidazole. Eight elution fractions were collected using 250 µL of lysis buffer containing 150 or 200 mM imidazole. The imidazole was then removed from the purified protein using 30 K (p53) or 10 K (p53C) Amicon Ultra centrifugal filters (Millipore) and completing the volume to 15 mL with 50 mM Tris pH 8, 150 mM NaCl and 5% glycerol. Centrifugation was performed at 3,700 g for 30 minutes. Protein concentration was measured using extinction coefficients of ε_280_=36035 cm^-1^M^-1^ (p53) or 18910 cm^-1^M^-1^ (p53C) determined with ProtParam [19]. Protein aggregation was induced by incubation at 42 °C for 24 h [20,21]. Labeling of p53 and p53C with DyLight 650 (Thermo Scientific) was carried out following the manufacturer’s instructions.

### Western blot

Following purification, 4 ng of recombinant p53 or p53C was denatured at 95 °C in SDS-PAGE sample buffer [0.5% SDS (w/v), 1,25% 2-mercaptoethanol (v/v), 4% glycerol (v/v), 0,01% bromophenol blue (w/v), 15 mM Tris–HCl, pH 6,8]. Proteins were either stained using Coomassie Blue or detected by western blot using anti-p53 1C12 (1:1000) (Cell Signaling) or anti-pentaHis (1:1000) (Qiagen) antibodies. For trypsin assays, 100 µL of DyLight 650-labeled p53 at 5 µM was incubated in 500 µL of either pre-warmed 0,25% trypsin/EDTA or phosphate-buffered saline (PBS) at 37 °C during 5 minutes. Digestion was stopped by placing the tubes on ice. 100 µL of diluted protein was precipitated using chloroform/methanol [22] and denatured at 95 °C in SDS-PAGE sample buffer.

### In vitro characterization of p53 aggregates

Binding of 8-anilino-1-naphthalenesulfonate (ANS) to p53 aggregates in PBS 20 mM was evaluated by measuring the fluorescence enhancement of 8-anilino-1-naphthalenesulfonate (10 µM) in the presence of 5 µM protein preparation upon excitation at a wavelength of 380 nm. The emission spectra were integrated from 400 to 600 nm.

Electron microscopy analyses were carried out as previously described [23].

### Cell culture

Human adenocarcinoma HeLa cells and mouse fibroblast NIH3T3 cells (ATCC) were cultured in DMEM medium supplemented with 10% fetal calf serum, penicillin/streptomycin 1X, sodium pyruvate 1 mM and amphotericin β 2,5 µg/mL in 5% CO_2_ humidified atmosphere at 37 °C. All reagents for cell culture were purchased from Wisent.

### Uptake of extracellular p53 aggregates and confocal microscopy

HeLa and NIH3T3 cells were plated on microscope coverslips in 24-well plates in 500µL medium. After 24 h, the cells were exposed to 1 µM of DyLight 650-labeled p53 aggregates with or without 50 µg/mL Dextran-488 (10 000 kDa, Invitrogen) overnight and then washed and incubated with 0,25% Trypsin/EDTA during 90 s to remove the excess of aggregates. Cells were fixed using 4% paraformaldehyde in PBS for 20 minutes at room temperature, washed once in PBS and permeabilized in PBS containing 0,15% Triton X-100 for 5 min. Cells were blocked in PBS containing 10% Normal Goat Serum (Wisent) for 20 min and then incubated 20 min with Phalloidin-Alexa 568 (Invitrogen). Cells were incubated for 5 minutes at room temperature with Hoechst (Sigma-Aldrich), then washed three times in PBS and mounted with SlowFade Gold (Invitrogen) onto microscope slides. Confocal analyses were carried out as previously described [24]. For time-dependent analysis of aggregate entry, HeLa cells were seeded in 6-well plates and DyLight 650-labeled p53 aggregates were added for 15 h, 6 h, 3 h, or 1 h before trypsinization. 50 000 cells were analyzed by flow cytometry (FACS Aria, BD Biosciences) for each time point.

### De novo- and co-aggregation of cellular p53 with recombinant p53 aggregates

NIH3T3 cells were plated on glass coverslips in 24-well plates in 500 µL medium. Transfection of p53^GFP^ construct or pEGFPN1 empty vector (Clontech) was performed using GeneCellin (BioCellChallenge) following the product manual. After 24 h, the cells were exposed to 1 µM recombinant p53 aggregates for 24 h and then fixed using 4% paraformaldehyde in PBS for 20 minutes at room temperature. Cells were washed once in PBS, incubated for 5 minutes at room temperature with Hoechst, then washed three times in PBS and mounted with Slowfade Gold onto microscope slides.

For co-aggregation experiments, HeLa cells were plated in 24-well plates in 500 µL medium. Cells were exposed to 1 µM of DyLight 650-labeled recombinant p53 aggregates for 24 h, treated with 0,25% trypsin/EDTA and re-plated onto glass coverslips. Cells were left to recover overnight then treated with 100 µM etoposide (Sigma-Aldrich) for 2 h. Cells were fixed using 4% paraformaldehyde, permeabilized for 5 min in 0,15% Triton X-100 (Bioshop) then stained with Sudan Black B (Sigma-Aldrich) as previously described [25]. Briefly, cells were incubated in 0,1% Sudan Black B in 70% ethanol for 20 minutes in a humid chamber. Next, cells were washed 3 times in PBS-tween 0,02% and a final wash was performed using PBS. Cells were incubated for 5 minutes at room temperature with Hoechst in PBS, then washed three times in PBS and mounted with Slowfade Gold onto microscope slides.

### Perturbation of endocytosis

All inhibitors were purchased from Sigma-Aldrich. HeLa cells seeded 100 000 cells/well in 6-well plates were incubated in 50 µM genistein, 5 µM chlorpromazine hydrochloride, 100 µM 5-*N*-ethyl-*N*-isopropyl-amiloride (EIPA), 25 µg/mL cytochalasin D, 5 mM methyl-β-cyclodextrin (MβCD) or vehicle in combination with 1 µM DyLight 650-labeled p53 aggregates and left for 3 h. Cells were trypsinized and analysed by flow cytometry. The effect of the dominant-negative (DN) mutant was determined by transfection of HeLa cells with dynamin K44A DN 24 h prior to exposure to DyLight 650-labeled p53 aggregates. 50 000 events were recorded per experiment and only single cell events were analysed.

### Statistical analyses

Data of p53 aggregates uptake were analyzed by a paired Student’s t-test. Data of de novo aggregation of soluble p53 with recombinant p53 aggregates were analyzed by a Chi-square test. All statistical analyses were performed using Prism 5 GraphPad software.

## Results

### WT p53 aggregates *in vitro*

**Figure 1:**
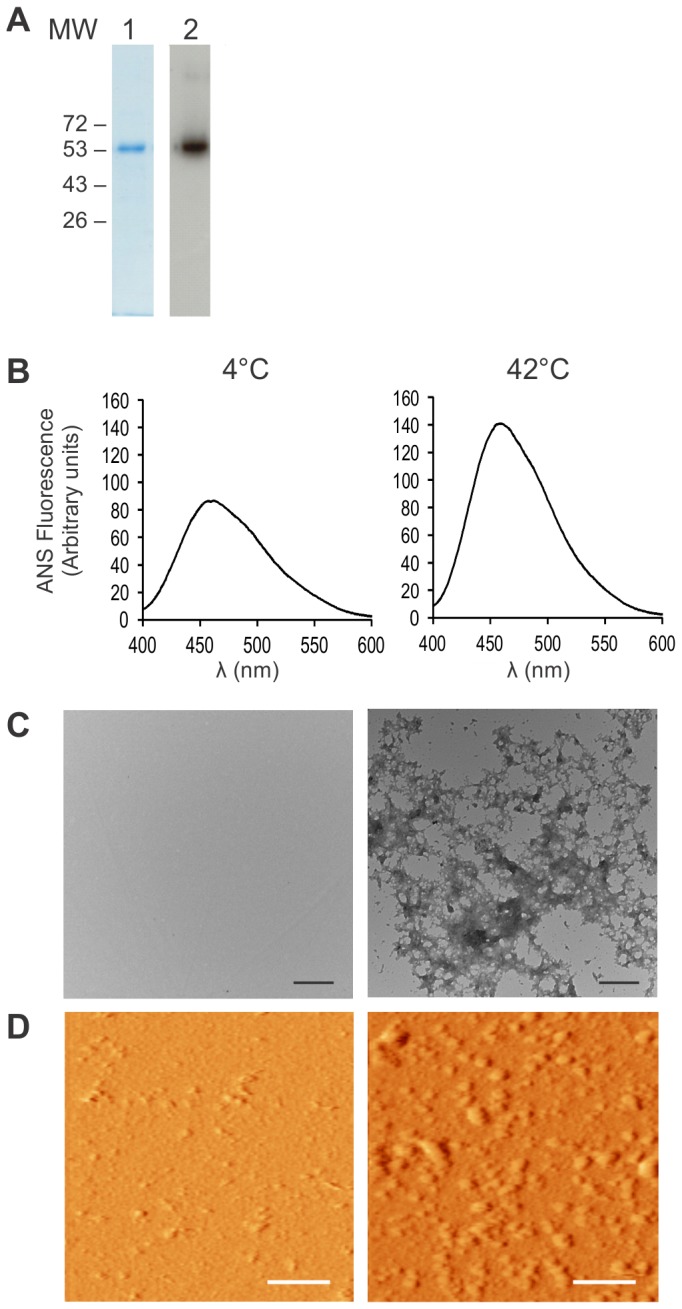
p53 forms amorphous aggregates at 42 °C. (A) Purified p53 protein were resolved by 10% SDS-PAGE and stained with Coomassie Blue (lane 1) or analyzed by Western Blot (lane 2). (B) 5 µM p53 protein was left at 4 °C (soluble) or heated at 42 °C (aggregates) for 24 h and analyzed by ANS fluorescence (C) Transmission electron microscopy showing mostly soluble (incubated at 4°C) or aggregated (incubated at 42°C) p53. (D) Atomic force microscopy showing mostly soluble (incubated at 4°C) or aggregated (incubated at 42°C) p53. Scale bars: 500 nm (C) and 1 µm (D).

Wild-type human p53 protein was expressed in bacteria, purified (Figure 1a) and aggregation was induced at 42 °C. The aggregates formed were characterized by their ability to bind 8-anilinonaphtalene-1-sulphonate, a fluorescent molecular probe used to determine conformational changes by binding to exposed hydrophobic regions of a protein [26]. An increase in fluorescence intensity, as seen in Figure 1b, indicates binding of the dye to hydrophobic regions of the aggregates. Observation of the aggregates by electronic microscopy revealed their amorphous nature (Figure 1c), as opposed to amyloid or fibrillar species seen for Aβ or α-synuclein [27,28]. Atomic force microscopy revealed the presence of bigger aggregates for p53 protein incubated at 42 °C compared to recombinant p53 protein left at 4 °C. Appearance of the aggregates using this technique was more granular, similar to aggregates of the p53 core domain seen previously [9].

### p53 aggregates enter HeLa and NIH3T3 cells

**Figure 2:**
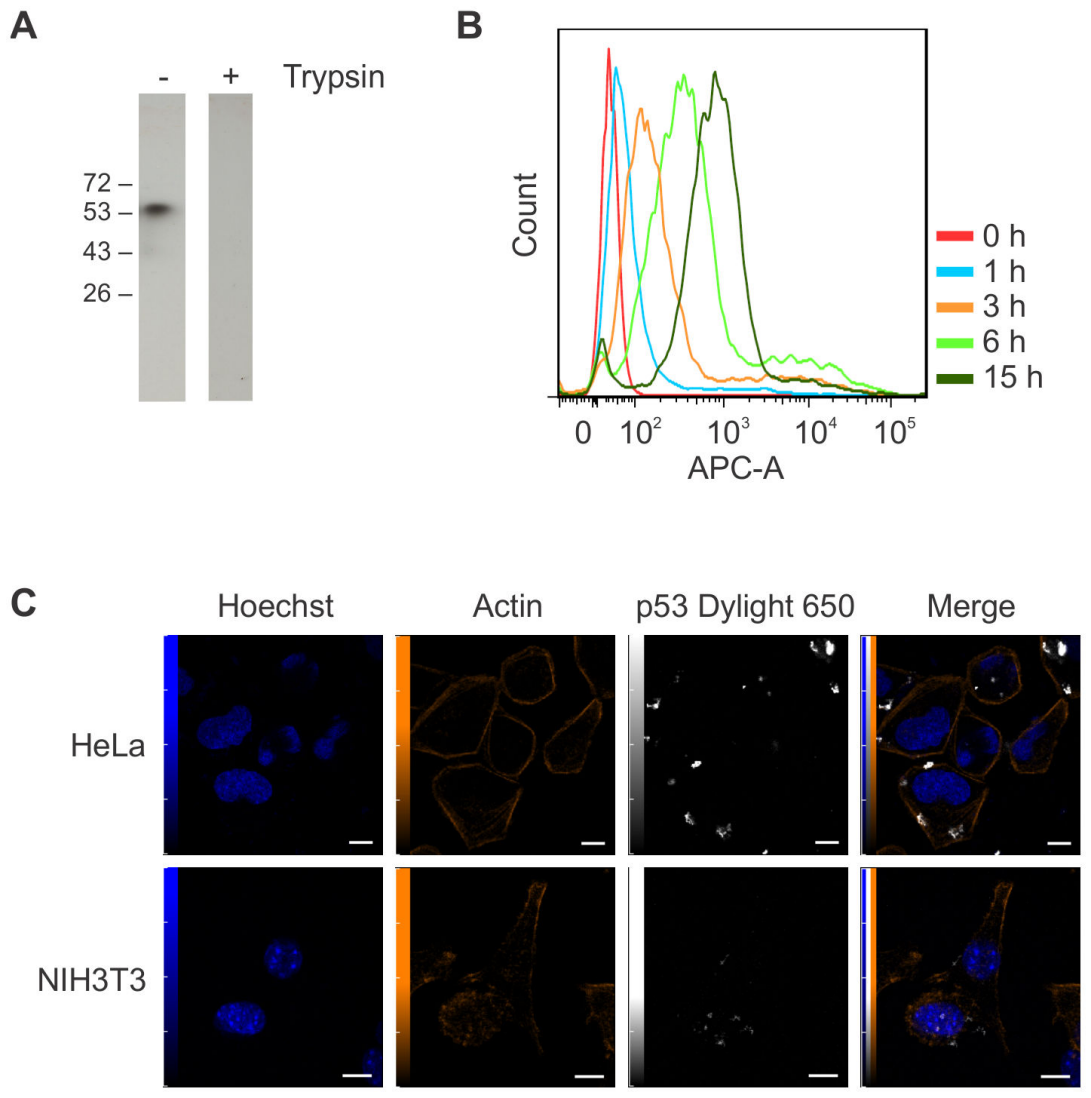
p53 aggregates are internalized by HeLa and NIH3T3 cells. (A) Aggregated p53 was treated with trypsin for 5 min, resolved by 10% SDS-PAGE and analysed by Western Blot. (B) HeLa cells were incubated with 1 µM DyLight 650-conjugated p53 aggregates for 24 h and analysed by flow cytometry. (C) HeLa and NIH3T3 were incubated with 1 µM DyLight 650-conjugated p53 aggregates for 24 h and visualized by confocal microscopy. Scale bars: 5 µm.

Similar to other protein aggregates [29–32], we investigated the possibility that p53 aggregates could also enter cells. HeLa cells incubated with DyLight 650-labeled p53 aggregates for different periods of time were treated with 0,25% trypsin/EDTA and sorted by FACS. Because p53 aggregates were discovered to be completely digested by 0,25% trypsin/EDTA within 5 minutes (Figure 2a), treatment with trypsin was used to remove extracellular aggregates, leaving intact only aggregates that have been internalized. As shown in Figure 2b, p53 aggregates enter HeLa cells in a time-dependent manner, with the majority of cells having internalized fluorescent aggregates after 15 h. Aggregate internalization was also visualized by confocal microscopy in two different cell lines using the same conditions (Figure 2c). HeLa and NIH3T3 cells were incubated with DyLight 650-labeled p53 aggregates for 24 h, treated with 0,25% Trypsin/EDTA and re-plated onto microscope slides in fresh medium overnight. Staining of actin with phalloidin was also used to visualize the cell contour and confirm that the aggregates are intracellular. Both cell lines internalized DyLight 650-labeled p53 aggregates with similar efficiencies.

### p53 aggregates enter HeLa cells via macropinocytosis

**Figure 3:**
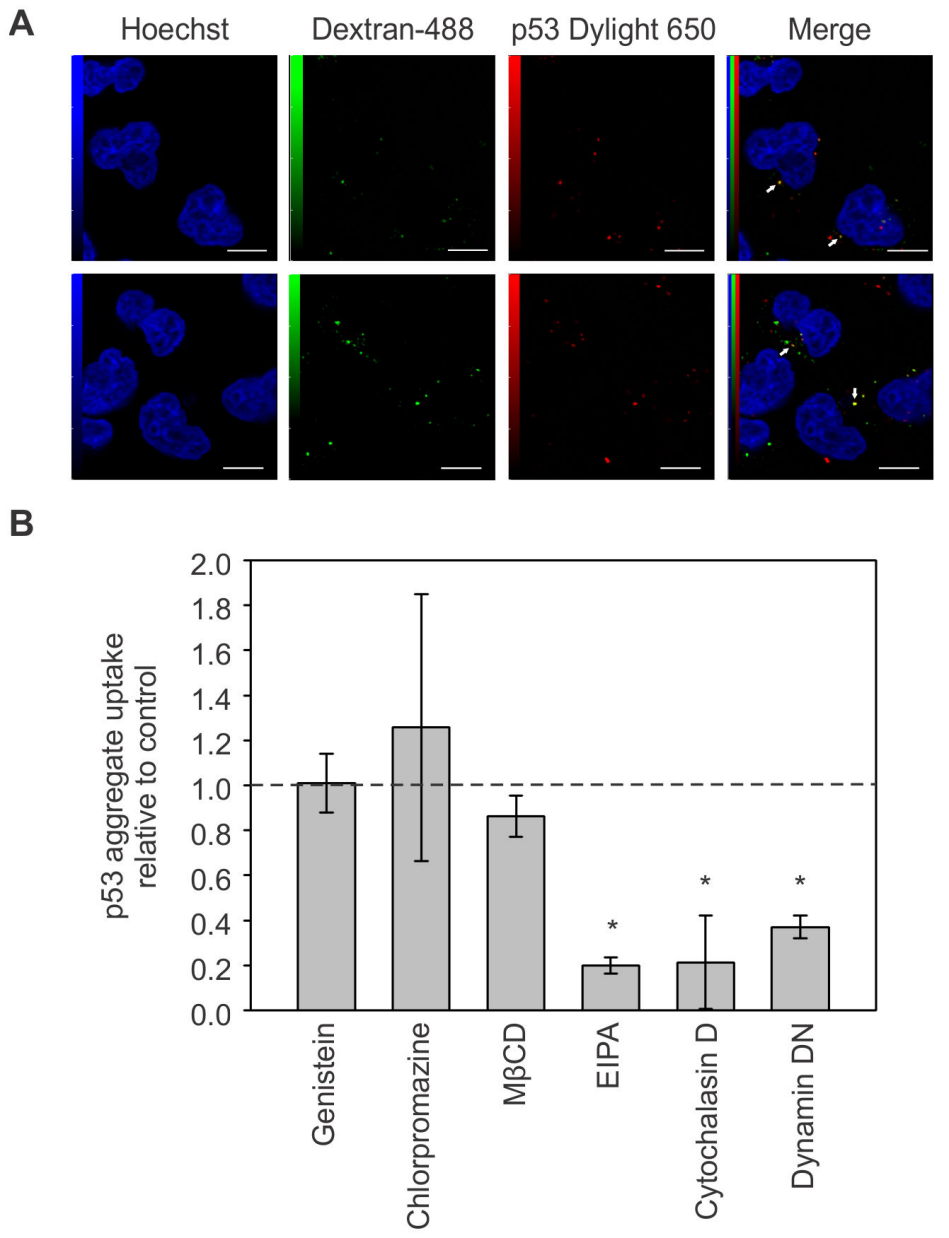
p53 aggregates are internalized via macropinocytosis. (A) HeLa cells were incubated with 1 µM DyLight 650-conjugated p53 aggregates and 50 µg/mL Dextran-488 overnight, and visualized by confocal microscopy. (B) HeLa cells were either treated with the indicated component or transfected with dominant-negative dynamin 24 h before incubation with 1 µM DyLight 650-conjugated p53 aggregates for 3h and analysed by flow cytometry. Mean value of 3 replicates is shown (*, p < 0,05); 50000 events were recorded per experiment. Scale bars: 10 µm.

We next examined the mechanism by which the aggregates penetrate HeLa cells by adding dextran-488, a marker of fluid-phase endocytosis, in the cell media along with DyLight 650-labeled p53 aggregates. Confocal microscopy shows a partial colocalization of dextran-488 with the aggregates (Fig. 3a, arrows). This finding indicates that p53 aggregates can enter cells via endocytosis. Thus, we used inhibitors of different endocytic pathways to pinpoint which ones contributed to the internalisation of p53 aggregates. We used chlorpromazine, an inhibitor of clathrin-coated pits, to determine whether p53 aggregates enter cells via a clathrin-dependent endocytosis [33]. Using flow-cytometry analysis, we determined that chlorpromazine did not affect internalization of DyLight 650-labeled p53 aggregates (Figure 3b). Similar results were obtained when cells were exposed to genistein, a tyrosine kinase inhibitor that inhibits caveolin-dependent endocytosis (Figure 3b). Lipid-raft dependent endocytosis inhibition with MβCD, which disrupts lipid rafts by extracting cholesterol from membranes [34], did not significantly affect internalization of aggregates by the cells. Exposure of cells to EIPA, an inhibitor of Na^+^/K^+^ pumps essential for macropinocytosis, however, greatly diminished entry of p53 aggregates into cells. The actin-polymerization inhibitor cytochalasin D produced a similar effect to EIPA, suggesting actin is an important component for internalization of p53 aggregates. Another potent inhibitor of p53 aggregate entry into cells was the dynamin dominant-negative mutant K44A (Figure 3b). Taken together, these results suggest macropinocytosis as the mechanism of entry of the p53 aggregates into HeLa cells, and that normal function of Na^+^/K^+^ pumps, dynamin and actin are needed for the internalization to occur.

### Recombinant p53 aggregates can induce aggregation of soluble p53

**Figure 4:**
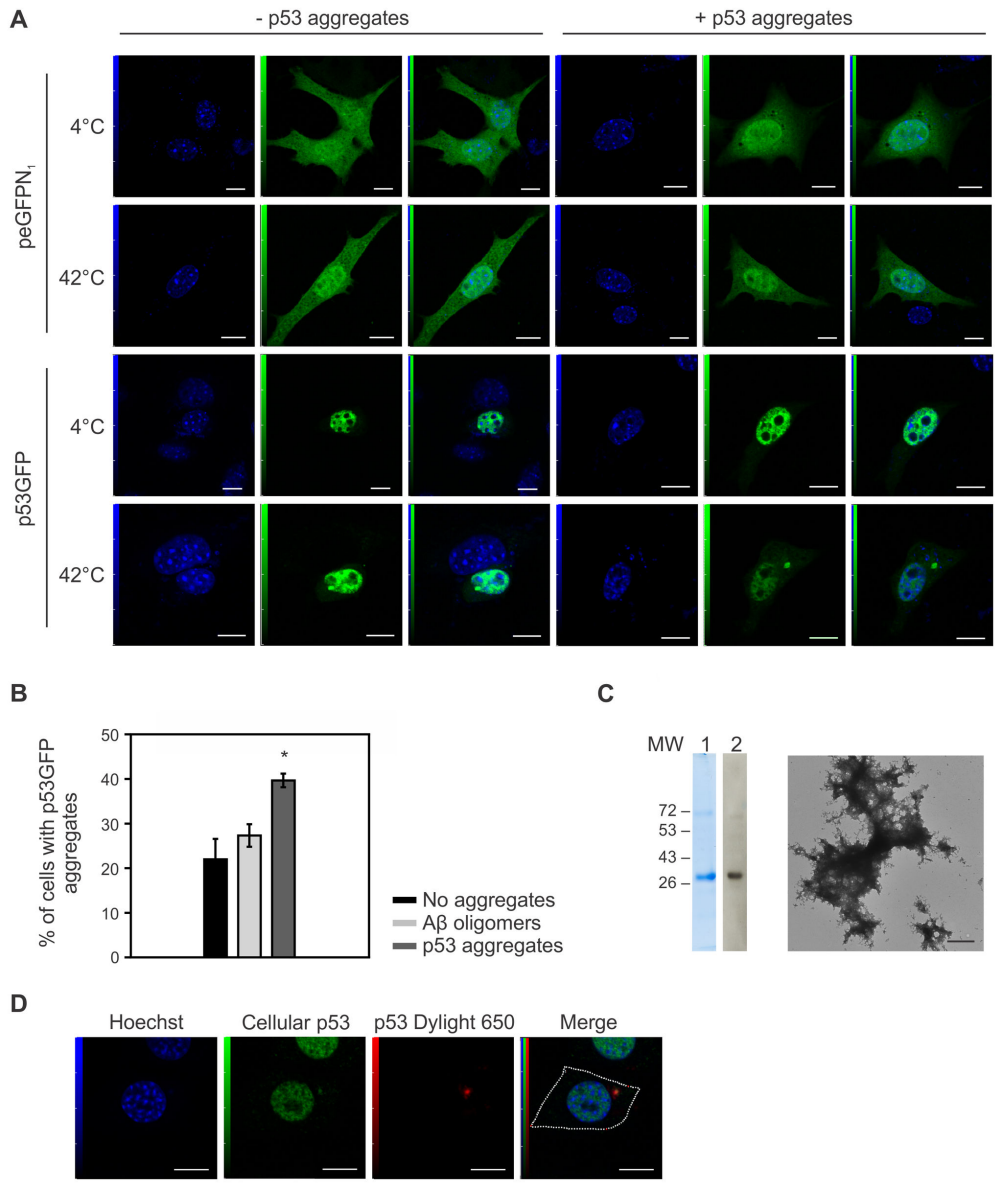
De novo aggregation of soluble p53 with recombinant p53 aggregates. (A) NIH3T3 cells were transfected with p53GFP or pEGFPN1 24 h prior to addition of 1 µM purified p53 protein incubated at 4 °C or 42 °C during 24 h. (B) % of cells containing p53GFP aggregates when incubated with either 1 µM p53 aggregates, Aβ oligomers or without aggregates. Mean value of 3 experiments is shown (*, p < 0,001); 100 cells counted per experiment. (C) Purified p53C protein was resolved by 10% SDS-PAGE and stained with Coomassie Blue (lane 1) or analyzed by Western Blot (lane 2). Transmission electron microscopy showing aggregated p53C. (D) NIH3T3 cells were incubated with 1 µM DyLight 650-conjugated p53C aggregates for 24 h, treated with 0,25% Trypsin/EDTA and re-plated onto microscope slides that were incubated with 100 µM etoposide for 2 h before visualization by confocal microscopy. Scale bars: 5 µm (A), 500 nm (B) and 10 µm (C).

We next investigated whether p53 aggregates could induce aggregation of soluble p53, similarly to PrP^c^ being recruited to form PrP^Sc^ aggregates. NIH3T3 cells were transiently transfected with p53^GFP^ or peGFPN1 (empty vector) and incubated with soluble or aggregated p53 for 24 h. Aggregation of p53^GFP^, but not GFP, was observed following incubation with p53 aggregates (Figure 4a). Furthermore, aggregation of p53^GFP^ was much more frequent in cells incubated with p53 aggregates than those incubated with soluble p53 (Figure 4a). To show that p53 aggregates are formed only if the cells are incubated with aggregates of the same protein, the p53^GFP^ or GFP-transfected cells were incubated with Aβ oligomers. The proportion of cells with p53^GFP^ aggregates was significantly higher in cells incubated with p53 aggregates than Aβ oligomers (Figure 4b). These results strongly suggest that only extracellular p53 aggregates can induce misfolding and aggregation of soluble p53^GFP^ in cells. To show direct contact between recombinant p53 aggregates and intracellular p53^GFP^, expression of endogenous soluble p53 was induced in HeLa cells using the topoisomerase inhibitor etoposide for 2 h following overnight incubation of the cells with DyLight 650-labeled p53C aggregates. p53C purification and aggregation were characterized by SDS-PAGE and electron microscopy, respectively (Figure 4c). Aggregates morphology observed by electron microscopy was found to be similar to that obtained for p53 full length (compare Figure 4c with Figure 1c). Incubation of the external aggregates with cells treated with etoposide and expressing soluble p53 led to co-aggregation, observed by revealing endogenous p53 with 1C12 anti-p53 antibody, whose epitope is located in the N-terminal part of p53 absent in truncated DyLight 650-p53C (Figure 4d). Together, these results show that exogenous p53C aggregates enter cells and induce intracellular aggregation of endogenously expressed p53.

## Discussion

Since half of all cancers harbour a WT p53 protein, we investigated whether p53 could form aggregates even in the absence of a structural mutation. In this study, we emphasize the shared characteristics between p53 and prionoids. The most notable features of prionoids are protein aggregation, internalisation of aggregates into cells, co-aggregation with the endogenous corresponding protein and cell-to-cell transfer of the aggregates.

The p53 protein has limited structural stability, due in part to the unstructured nature of its N-terminal domain [7] not unlike PrP and α-synuclein [35,36]. This feature is thought to facilitate the aggregation of p53, which has been carried out previously using heat, acidic pH and high pressure [8,9,16,37]. Nevertheless, many of these earlier studies only use specific domains of the protein for aggregation studies; the aggregation of the full-length protein has only received limited attention, despite its possible involvement in cancer.

Many previous studies show internalization of aggregates such as aggregated PrP, tau and expanded polyglutamine [30–32]. However, this is the first time that p53 aggregate entry into cells has been shown. Colocalization of dextran-488 with the aggregates (Figure 3a, arrows) indicates that p53 aggregates can enter cells via endocytosis, similarly to PrP and other prionoid aggregates, such as Tau and SOD1 [29–31]. Despite this finding, one cannot eliminate the possibility that p53 aggregates penetrates the cell by direct diffusion through the plasma membrane, similarly to polyglutamine aggregates [32].

The different drugs used to perturb endocytosis confirmed its involvement in p53 aggregate internalization, as p53 entry strongly depends on dynamin I activity and is greatly affected by EIPA, a Na+/H+ ion pump inhibitor. In fact, the dynamin-dependent and EIPA-sensitive aspects of p53 aggregate internalization are reminiscent of macropinocytosis-like mechanisms involved in bluetongue virus-1 and Ebola virus uptake [38]. The size of intracellular p53 aggregates (> 1 µm) as seen in Figure 2 not only strengthens the possibility that macropinocytosis is involved in their internalization but also eliminates the possibility that aggregates enter cells via clathrin- or caveolin-coated pits. In fact, clathrin or caveolin-coated vesicles are approximately 120 and 60 nm in size, respectively, whereas macropinosomes vary in size from 0,2 to 5 µm [39,40]. Although genistein and chlorpromazine had no significant effect on p53 aggregate entry into cells, clathrin- and caveolin-dependant mechanisms of entry cannot be eliminated as a possible secondary entry mechanism for p53 aggregates, because small aggregates may not have been detected by FACS analyses.

Until now, co-aggregation of exogenous p53 aggregates with soluble p53 has only been shown *in vitro* with the use of a known mutant of p53C, p53 R248Q [16]. In the present study we not only show the formation of aggregates using WT p53, but also that these aggregates can recruit cellular WT p53 to form *de novo* aggregates within the cells. The co-aggregation of protein aggregates and their corresponding protein *in cellulo* is found to be a key feature in the propagation of prion-like protein aggregates, such as TDP-43, α-synuclein, SOD1, tau and Huntingtin [29,31,32,41,42]. Finally, in this study we emphasize the importance of using the full-length p53 protein in aggregation studies relating to cancer, as less than 10% of cancers harbour a truncated form of the protein [43]. Thus, full-length p53 must be used to mimic physiological conditions as accurately as possible.
